# Evaluation of Fracture Strength of Fiber-Reinforced Direct Composite Resin Restorations: An In Vitro Study

**DOI:** 10.3390/polym14204339

**Published:** 2022-10-15

**Authors:** Nassreen Hassan Mohammad Albar, Waad Fahmi Khayat

**Affiliations:** 1 Department of Restorative Dentisrty, College of Dentistry, Jazan University, Jazan 45142, Saudi Arabia; 2 Department of Restorative Dentistry, College of Dentistry, Umm Al-Qura University, Makkah 24381, Saudi Arabia

**Keywords:** composite resin, fracture strength, polyethylene, ribbond

## Abstract

This in vitro study aimed to compare the fracture strength of direct non-reinforced class II composite resin restorations and polyethylene fiber-reinforced restorations, and also to investigate the influence of the locations of polyethylene fibers within the cavity on the fracture strength. Sixty freshly extracted human teeth were disinfected and prepared (class II cavity design). The teeth were assigned randomly into four groups (n = 13). Group I (control) was restored with nano-hybrid composite resin. The other three experimental groups were restored with the same composite resin material reinforced by polyethylene fibers (Ribbond) at different locations. Fibers were placed either on the axial wall (Group II), on the gingival floor (Group III), or on the axial wall and pulpal/gingival floor (Group IV) of the proximal cavity. All the teeth were subjected to thermocycling to simulate the oral environment. The fracture strength was measured using a universal testing machine. Group IV had the highest mean fracture strength at maximum load (148.74 MPa), followed by Group II (140.73 MPa), Group III (136.34 MPa), and Group I (130.08 MPa), with a statistically significant difference from the control group (*p* = 0.008) but not between groups II and III.

## 1. Introduction

Dental caries are one of the most prevalent chronic diseases worldwide [1]. Despite preventive measures and campaigns, the need for dental restorations continues to rise. Traditionally, direct restorations are used as a conservative approach to restore teeth [2]. Composite resin restorations are generally the first choice in anterior [3,4] and posterior restorations [5] due to their aesthetic appearance, conservative cavity preparation designs, and relatively lower cost [5]. It is estimated that almost a billion composite resin restorations are placed every year across the globe [6,7]. Despite their popularity, composite resin restorations have a failure rate of approximately 5% based on wear and fractures [8,9,10].

Removal of large amounts of the tooth structure may result in a weakened restored tooth. Class II restorations are more prone to fracture due to the involvement of the marginal ridge, the higher stresses in the wide isthmus area, and the wedging effect causing horizontal stresses that weaken the cavity walls and lead to fractures [11,12,13,14]. Because fracture of the restoration remains the primary mode of failure in large restorations, several strategies have been adopted to try to minimize these shortcomings [15,16]. These strategies to improve the strength of composite resin materials include but are not limited to reinforcement of materials by different means, such as nano-fillers or polyethylene fibers. Fibers can be either embedded into the resin matrix during the manufacturing of the material, to achieve better physical and mechanical properties, or externally applied during the placement of direct restorations.

Polyethylene is a modern material that has stood the test of time. Chemically, it is a thermoplastic polymer that is used universally in industry and manufacturing. It forms the basis of high-strength reinforced manufacturing in aircraft doors, safety equipment, and building construction materials [17]. In dentistry, it has several applications, including endodontic posts and cores, space maintainers, splinting, and in fixed and removable prosthodontics [18,19]. Polyethylene fibers are made of aligned polymer chains with a low modulus of density, allowing for greater impact resistance [20]. The leno weave ultra-high modulus (LWUHM) polyethylene fiber offers an opportunity to improve the performance of existing materials. This particular class of polyethylene fiber is a reinforced ribbon whose fiber architecture enables even force distribution and improved mechanical properties [21]. Numerous procedures utilize FRC, such as the fabrication of single crowns, full and partial coverage-fixed partial dentures, and periodontal splints [22].

Fractural strength is a vital characteristic of any restorative material. It indicates the resistance of a material to cracking and resistance to fracture [23]. Reinforcement of composite resin materials with fibers enhances the fracture resistance of the material and improves its flexural strength [24,25,26]. From a clinical point of view, this biomimetic approach is considered non-invasive compared with indirect restorations, cost effective, time efficient, and a promising technique to protect against marginal fracture of large restorations. Although many studies have examined polyethylene-fiber reinforced composites, there are only few published studies on the fracture strength of polyethylene-fiber reinforced class II composite resin restorations.

Therefore, the aim of the study was to compare the fracture strength between direct non-reinforced class II composite resin restorations and polyethylene fiber-reinforced restorations, and also to investigate the influence of the locations of polyethylene fibers applied within the cavity on the fracture strength. To our knowledge, none of the currently published studies have investigated the influence of the location of the fibers on the mechanical properties of large composite restorations.

## 2. Materials and Methods

The study protocol was approved by the Institutional Review Board (IRB) of the College of Dentistry, Jazan University, Jazan, Saudi Arabia (Ref: REC-43/08/181).

### 2.1. Sample Size Calculation

A sample size calculation was conducted using G Power (Version 3.1). Based on Patnana et al. [27], a mean of 169.28 was assumed for Group A, a mean of 255.71 was assumed for Group B, and it was also assumed that, compared to Group B, the mean of Group C would be the same. The mean of Group D would be 20% higher than the latter groups. A typical standard deviation of 50 was assumed. Based on these assumptions, a sample size of n = 12 per group was adequate to obtain a Type I error rate of 5% and a power of 80%, with an effect size of 0.5.

### 2.2. Study Design and Specimens Preparation

This study was an in vitro experimental design—a collection of 60 freshly extracted intact human premolars/molars from various dental clinics were cleaned and disinfected. The teeth were disinfected by immersing them in 0.5% sodium hypochlorite solution for 15 min. Distilled water was the storage media for the sterilized teeth. Inspection of teeth determined they were intact with no extensive wear, caries, cracks, fractures, or previous restorations. The selected teeth also had comparable dimensions and morphology. Teeth were mounted into a resin block to a level of 2 mm apical to the cementoenamel junction. A silicon index (Aquasil Soft Putty, Dentsply, Charlotte, NC, USA) was fabricated (covering the occlusal third of the tooth) for the occlusal surface of each tooth to assess in restoring the occlusal anatomy during the restoration placement. The proximal surface was restored following the anatomical contour of the tooth structure using a matrix band.

A standardized class II occlusodistal cavity was prepared with an occlusal cavity depth of 2 mm and buccolingual (isthmus) width of one-third of the inter-cuspal distance on each tooth. The facial and lingual walls were parallel with a cavosurface angle of 90°. All internal line angles were rounded. The width of the proximal box was one-third of the total facio-lingual distance of the marginal ridge. The axial depth of the proximal cavity was 1.5 mm, and the gingival floor was seated 1 mm coronal to the cementoenamel junction with margins on the enamel [28]. The extension of the cavity outline on the predetermined extension was drawn with a water-resistant pencil. The cavities were prepared by a fissure carbide bur using a high-speed handpiece and water coolant by the same dentist on all teeth. The cavity outline and depth were checked with a periodontal probe and caliper to ensure standardization. The prepared teeth were randomly assigned to one of four groups.

- Group I: Restored conventionally with direct composite resin restoration.
- Group II: Restored with composite resin restoration reinforced by polyethylene fibers placed on the axial wall of the proximal cavity.
- Group III: Restored with composite resin restoration reinforced by polyethylene fibers placed on the gingival floor of the proximal cavity.
- Group IV: Restored with composite resin restoration reinforced by polyethylene fibers placed on both the axial wall and the pulpal/gingival floor of the proximal cavity.

Group I was the control group. All the cavities in the control group were restored conventionally using composite resin (Filtek Z 250, 3M ESPE), following the manufacturing instructions for bonding with an Etch and Rinse (Ambar Universal, FMG Dental Group, Fort Lauderdale, FL, USA). The proximal wall was restored using an automatrix (Pro-Matrix Wide, Medicom, Montreal, QC, Canada) to the level of the junction of the middle and the occlusal third of the wall (determined by periodontal probe). The adaptation of the silicon index was checked for proper seating before proceeding. Then, the composite material was layered into the cavity. The occlusal print surface of the index was lightly wetted with Ceramage modeling liquid (Shofu, Kyoto, Japan). Then, the occlusal surface was restored using the silicon index as a stamp to establish the original anatomy of the occlusal part of the tooth structure and cured as recommended in the instructions. The surface was checked for voids or defects before and after curing. The experimental groups (II, III, IV) were restored by composite resin reinforced by polyethylene fibers (Ribbond Inc., Seattle, WA, USA). The bonding protocol was applied according to the manufacturer’s instructions (Ambar, FMG Dental Group, Fort Lauderdale, FL, USA). Then, a 0.5 mm layer of flowable composite resin (Opallis Flow, FMG Dental Group, Fort Lauderdale, FL, USA) was placed into the cavity. The MD length of the cavity was measured using a periodontal probe. A piece of fiber was cut to the measured length, wetted with unfilled bonding resin, and embedded into the composite resin layer (0.5 mm away from the external margin of the tooth structure) on the wall, floor, or both, according to the assigned group. Then, incremental layers of composite resin restoration were used to fill out the rest of the cavity. The last layer was placed using the silicon index to establish the original anatomy of the occlusal part of the tooth structure and cured as per the instructions. All the specimens were finished and polished using the 2-step rubber spiral wheel (Sof-Lex Diamond Polishing System, 3M) according to the protocol recommended by the manufacturer, and then stored in distilled water at a temperature of 37 °C.

**Figure 1:**
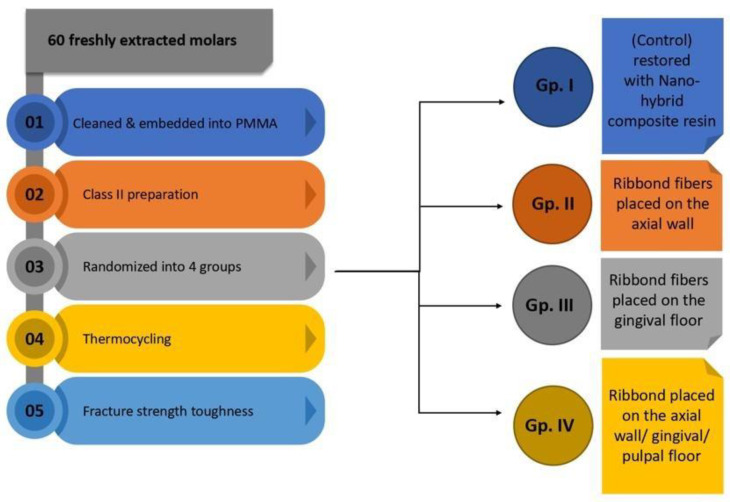
Graphical illustration of the study design.

To simulate the functioning of the oral cavity, teeth were subjected to thermocycling (Thermocycler THE-1100, SD Mechatronik, Feldkirchen-Westerham, Germany) for 10,000 cycles at 5 °C and 55 °C, with each cycle corresponding to a 15 s bath at each temperature and transfer time of 5 s (ISO Recommendations 11405:1994) [8,29] (Figure 1).

### 2.3. Evaluation of the Fracture Strength

**Figure 2:**
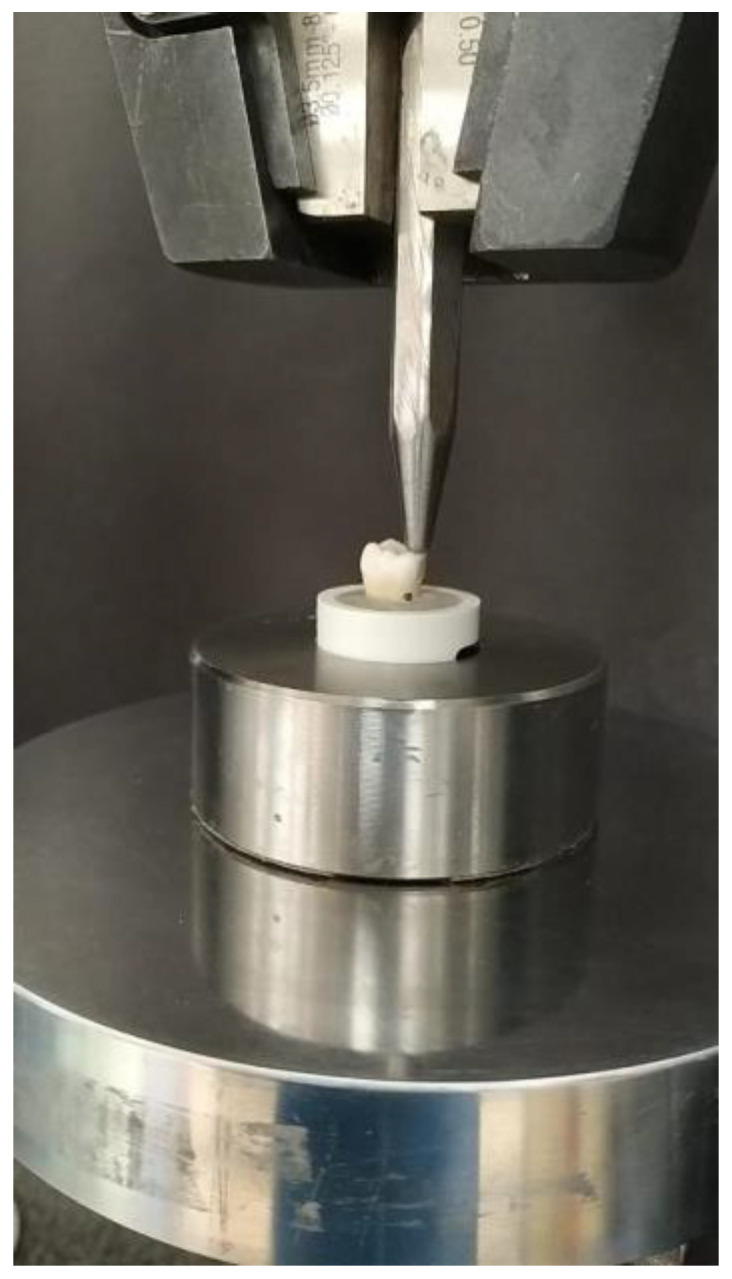
Fracture strength test by universal testing machine using a 3 mm diameter steel ball contacting the restoration at the isthmus area at a crosshead speed of 0.5 mm/min until fracture.

The fracture strength was measured using a universal testing machine (Instron model-5965). The roots of the restored molars were embedded in polymethyl methacrylate resin (PMMA) 1 mm below the cement enamel junction, and PVC (polyvinyl chloride) pipes were used as molds to hold the material. The molar was placed under the Instron universal testing machine with the load cell placed perpendicular to the proximal box of the composite restoration. Each specimen was subjected to axial compressive load or 1000 N to fracture using a 3 mm diameter steel ball contacting the restoration at the isthmus area, at a crosshead speed of 0.5 mm/min [9]. The maximum loading force was registered in newtons (N) until fracture occurred (Figure 2). The settings were controlled and the results were displayed using the BlueHill software 3.22.1373. Norwood, MA, USA. The fracture strength was calculated as follows:

Fracture Strength (MPa) = Maximum load (N)/Surface Area (mm^2^)

### 2.4. Statistical Analysis

The outcome variable assessed was the fracture strength. The normality was assessed using the Shapiro–Wilk test. If the assumptions of normality were satisfied, means and standard deviations were reported. Comparisons of the groups were made using a one-way analysis of variance (ANOVA). A post hoc analysis was performed using the Tukey–Kramer HSD test for statistically significant results found by ANOVA. All *p*-values less than 0.05 were considered statistically significant. All the analyses were performed using the Excel 365 (Microsoft Office) and SPSS software, v.23 (IBM Corp., New York, NY, USA).

## 3. Results

**Figure 3:**
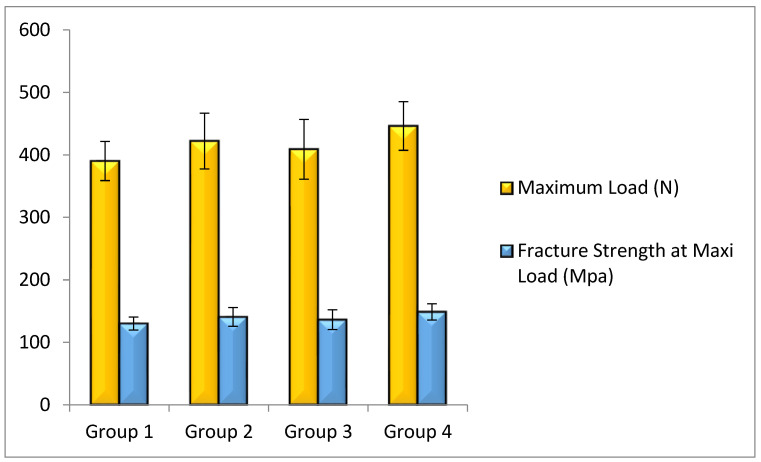
Means of fracture strength and maximum load of each group.

The distribution of data in tests for maximum load and fracture strength for all groups had a normal distribution according to the Shapiro–Wilk test. Means and standard deviation of the reported results are shown in Figure 3.

Group IV (polyethylene fibers placed on both the axial wall and the pulpal/gingival floor of the proximal cavity) sustained the highest mean maximum load (446.21 N) and presented the highest fracture strength at maximum load (148.74 MPa) of all the groups. This was followed by Group II (polyethylene fibers placed on the axial wall of the proximal cavity), in which the mean maximum load and fracture strength at maximum load were 422.18 N and 140.73 MPa, respectively. Group III (comprised of restorations with polyethylene fibers placed on the gingival floor of the proximal cavity) sustained a maximum load of 409.01 N and fracture strength at maximum load of 136.34 MPa. Group I (the control group) presented a maximum load of 390.23 N and a fracture strength of 130.08 MPa. The one-way ANOVA showed a statistically significant difference in the Mean Maximum Load and Fracture Strength at Maximum Load between all the groups (*p* = 0.012). There was a statistically significant difference between Group IV and Group I (*p* = 0.008). However, the differences of fracture strength between the remaining groups were not statistically significant (Table 1).

## 4. Discussion

The primary outcome of this study was obtaining the fracture strength of the reinforced nano-hybrid composite resin restorations with polyethylene fibers that were placed at different locations within class II cavities, compared to non-reinforced nano-hybrid composite resin restorations. The study found that restorations reinforced with polyethylene fibers on both the axial wall and gingival/pulpal floor showed statistically significant higher load-bearing capacity and fracture strength than the non-reinforced restorations. This study indicated that restorations in Class II cavities can be strengthened significantly by adding polyethylene fibers to the axial wall and the pulp/gingival floor of the proximal cavity, as compared to restorations not reinforced with fibers. In the present study, there was no statistically significant difference between the results of Group 2 and Group 3, indicating that the position of the fibers does not affect the outcome if the fibers are used on a single surface.

Modern direct restorative materials must provide aesthetics, adequate functionality, and protection of the remaining sound tooth structure. Loss of dentin in posterior teeth makes it difficult to reinforce the remaining tooth structure and restoration. The loss of one marginal ridge accounts for a mean loss of 46% in relative cusp stiffness [30].

Ribbond has been commercially available since 1992. The leno woven polyethylene fibers allow for close adaptation to the contours of the tooth [31]. The fiber layer increases the load-bearing capacity of restorative material and inhibits crack propagation [32]. For their application, the fibers must be saturated with wetting resin and then introduced into the flowable resin. It is believed that the leno design improves the penetration of the wetting resin, hence enhancing the chemical bond between the fibers and the flowable resin, forming a single amalgamated structure [33]. Additionally, the manufacture of this material uses cold gas plasma, which results in high adhesion to composite resins [34].

The high modulus of elasticity and low flexural modulus of polyethylene fibers modify the stresses at the interface formed by etched enamel and resin [35]. Polyethylene fibers embedded in flowable resin under composite restoration increase the fracture strength and micro-tensile bond strength to dentin [21,31]. The density of the fixed nodal intersections of low-weight ultrahigh molecular weight fibers maintains the integrity of the restoration and transfers the stresses in the bulk of the restoration efficiently along well-defined paths. Some studies have reported improved mechanical properties and that this can probably be attributed to transferring stress from the polymer matrix to the fibers [36].

Belli S. et al. claimed that the lock-stitch pattern transfers the forces through the fibrous weave without propagating the stress into the resin [36]. Flexural force on composites causes rapid propagation of cracks on the tensile face that result in failure in the absence of fiber reinforcement. Placement of fibrous ribbon provides interfaces that result in inhibition of crack propagation. When a crack occurs, the interwoven fibers blunt the path of propagation and redirect it along the weaker interface [37].

The results of the current study are in accordance with a study by Ayad et al., where they found that Class II cavities filled with fiber-reinforced restorations had higher fracture strength compared to the cavities restored with conventional (unreinforced) resin composites. The difference was about 39.7% in their study [38]. However, in contrast to ours, their study did not investigate the influence of the location of the fibers within the cavity.

Though the materials were tested in vitro, thermocycling was carried out before testing for strength. Simulating the intraoral temperature changes, thermocycling induces artificial aging of materials. This study also provides data as a part of value-based dental research which can help in evidence-based dentistry and improve treatment outcomes. Intraorally, the forces produced differ in magnitude, speed, and direction. This cannot be adequately replicated in a laboratory setting. Our application of force in a single direction at a constant speed could be a limitation in our study. Future studies could employ cyclic and shear forces that simulate the masticatory cycle of forces experienced intraorally. Furthermore, only a single type of fiber and resin composite was evaluated. Further evaluation of mechanical, chemical, and thermal stresses should be undertaken on the durability of the restoration. Clinical studies are required to confirm and validate the results of the present study.

## 5. Conclusions

Within the limitations of the study and based on our findings, the reinforcement of class II direct composite resin restorations with polyethylene fibers increased the fracture strength of the restorations in comparison with non-reinforced restorations. The increase was statistically significant compared to the non-reinforced restorations only when fibers were applied to the axial, gingival, and pulpal inner surfaces of the cavity; placing the polyethylene fibers underneath the composite resin restoration along both the axial wall and pulpal/gingival floor resulted in higher fracture strength than applying the fibers on either the axial wall or gingival floor, but the difference was not statistically significant.

## Figures and Tables

**Table 1:** Comparison of the Mean Fracture Strength at Maximum Load between the groups.

		N	Mean	SD	*p* Value
Fracture Strength at Max Load (Mpa)	Group 1 ^b^	12	130.08	10.45	0.012
	Group 2 ^a,b^	13	140.73	14.90	
	Group 3 ^a,b^	13	136.34	15.93	
	Group 4 ^a^	13	148.74	12.97	

ANOVA statistical analysis was performed to compare means at *p* < 0.05. Post hoc test was used to determine the significantly different groups. Identical letters correspond to a lack of statistical significance, whereas different letters indicate a statistically significant difference at *p* < 0.05.

## Data Availability

All data underlying the results of the current study are available as part of the article. No additional sources or data are required.
